# Investigating the Effect of Different Treatments with Lactic Acid Bacteria on the Fate of *Listeria monocytogenes* and *Staphylococcus aureus* Infection in *Galleria mellonella* Larvae

**DOI:** 10.1371/journal.pone.0161263

**Published:** 2016-09-12

**Authors:** Athena Grounta, Paschalis Harizanis, Eleftherios Mylonakis, George-John E. Nychas, Efstathios Z. Panagou

**Affiliations:** 1 Laboratory of Food Microbiology and Biotechnology, Department of Food Science and Human Nutrition, Agricultural University of Athens, Athens, Greece; 2 Laboratory of Sericulture and Apiculture, Faculty of Crop Science, Agricultural University of Athens, Athens, Greece; 3 Infectious Diseases Division, Warren Alpert Medical School of Brown University, Providence, Rhode Island, United States of America; Universitat Ulm, GERMANY

## Abstract

The use of *Galleria mellonella* as a model host to elucidate microbial pathogenesis and search for novel drugs and therapies has been well appreciated over the past years. However, the effect of microorganisms with functional appeal in the specific host remains scarce. The present study investigates the effect of treatment with selected lactic acid bacteria (LAB) with probiotic potential, as potential protective agents by using live or heat-killed cells at 6 and 24 h prior to infection with *Listeria monocytogenes* and *Staphylococcus aureus* or as potential therapeutic agents by using cell-free supernatants (CFS) after infection with the same pathogens. The employed LAB strains were *Lactobacillus pentosus* B281 and *Lactobacillus plantarum* B282 (isolated from table olive fermentations) along with *Lactobacillus rhamnosus* GG (inhabitant of human intestinal tract). Kaplan-Meier survival curves were plotted while the pathogen’s persistence in the larval hemolymph was determined by microbiological analysis. It was observed that the time (6 or 24 h) and type (live or heat-killed cells) of challenge period with LAB prior to infection greatly affected the survival of infected larvae. The highest decrease of *L*. *monocytogenes* population in the hemolymph was observed in groups challenged for 6 h with heat-killed cells by an average of 1.8 log units compared to non challenged larvae for strains B281 (*p* 0.0322), B282 (*p* 0.0325), and LGG (*p* 0.0356). In the case of *S*. *aureus* infection, the population of the pathogen decreased in the hemolymph by 1 log units at 8 h post infection in the groups challenged for 6 h with heat-killed cells of strains B281 (*p* 0.0161) and B282 (*p* 0.0096) and by 1.8 log units in groups challenged with heat-killed cells of LGG strain (*p* 0.0175). Further use of CFS of each LAB strain did not result in any significant prolonged survival but interestingly it resulted in pronounced decrease of *L*. *monocytogenes* in the hemolymph at 24 h and 48 h after infection by more than 1 log unit (*p* < 0.05) depending on the strain. The results of the present work support the broader use of *G*. *mellonella* larvae as a low cost *in vivo* tool for screening for probiotic properties.

## Introduction

Probiotic microorganisms are defined as “live microorganisms which when administered in adequate amounts confer a health benefit on the host” [1]. A number of health promoting properties have been reported in the literature due to intake of probiotics, such as maintenance of the balance of the intestinal biota, alleviation of lactose intolerance, allergy prevention as well as anti-carcinogenic, antihypertensive and immunomodulatory properties [2,3,4,5,6]. Microorganisms regarded as probiotics are mainly lactic acid bacteria (LAB) belonging to *Lactobacillus* and *Bifidobacterium* genera that can be found in a variety of dairy products such as yogurt, cheese and fermented milk. However, due to the increasing number of consumers with different nutritional habits (e.g., veganism) or consumers with milk protein allergy, there is need for the development of non-dairy probiotic products [7]. Table olives, as plant based fermented products, have been reported as a promising alternative for their use as biological carriers of health promoting microorganisms originating from human gastrointestinal tract [8,9]. Further on, a number of recent studies has been undertaken for the isolation and characterization of novel strains originating from the olive’s autochthonous microbiota to be used as probiotics and as starter/protective cultures in table olive processing [10,11,12,13,14,15]. Although these studies were undertaken *in vitro* their results are promising and indicate that olives are a rich pool of probiotic candidates giving rise to the need for further investigation under *in vivo* conditions.

The majority of reported studies highlighting the beneficial properties of probiotic microorganisms has been conducted using mammalian hosts and predominantly mice. The mouse model has long been used in research as *in vivo* tool, as it provides a similarity to human anatomy, immune response and in some cases microbial susceptibility. However, invertebrates are considered good alternatives to mammalian hosts to study microbial pathogenesis since many aspects of their physiology are evolutionary conserved, while they can be obtained and maintained inexpensively without dealing with the ethical issues associated with mammalian studies [16]. The most representative invertebrates as model hosts are the amoebae (*Acanthamoeba castellanii* and *Dictyostellium discoideum*), the soil-living nematode *Ceanorhabditis elegans* and insect species including the fruit fly *Drosophila melanogaster*, the greater wax moth *Galleria mellonella* and the silk worm *Bombyx mori*.

In probiotic related research, there are few studies using non-mammalian hosts. Studies on the nematode *C*. *elegans* have shown that a newly isolated *Lactobacillus* spp. and bifidobacteria may pose beneficial effect in the life and health span of the worm [17,18], whereas feeding *C*. *elegans* with *L*. *acidophilus* NCFM has been shown to protect against the Gram positive pathogens *Enterococcus faecalis* and *Staphylococcus aureus* [19]. Further on, symbiotic lactobacilli appear to be involved in intestinal development and stem cell proliferation in the fruit fly [20]. More recently, *L*. *acidophilus* ATCC 4356 was found to attenuate the experimental candidiasis in the greater wax moth [21], while in another work eugenol in combination with LAB supernatants attenuates the virulence of *Listeria monocytogenes* in the same host [22].

Among the abovementioned invertebrate models, *G*. *mellonella* larvae are regarded as emerging models posing advantages over other invertebrates. The most important advantage is their ability to survive up to 37°C to which human pathogens have been adapted. Larvae are commercially available, inexpensive to purchase and easy to maintain in the lab. Their size ranges between 1.5–2.5 cm which is large enough to handle and deliver a precise amount of microbial load directly in the haemocoel by injection through the last proleg. Moreover, results can be quickly obtained since a large number of larvae can be inoculated within a short period of time [23], allowing thus high throughput experiments for assessing microbial virulence or testing antimicrobial compounds.

Most importantly, a positive correlation between virulence and host response has generally been found in *G*. *mellonella* and mammalian hosts for a range of microorganisms, such as *Acinetobacter baumanii* [24], *Pseudomonas aeruginosa* [25,26], *Yersinia pseudotuberculosis* [27], *Staphylococcus aureus* [28], *Listeria monocytogenes* [29,30], *Enterococcus faecalis* [31], *C*. *albicans* [23,32] and *Cryptococcus neoformans* [33]. It has also been observed that pre-exposure of *G*. *mellonella* to sublethal doses of pathogens or physical stress gives them the opportunity to sense and mount their humoral and cellular defence against invasion, protecting them from a subsequent pathogenic invasion [34,35,36]. A part of defence peptide repertoire of *G*. *mellonella* has been described. Cytrynska et al. [37] purified eight different peptides from larval hemolymphs immunized with non pathogenic *E*. *coli* D31. Five of them, Gm proline-rich peptide 2, Gm defensin-like peptide, Gm anionic peptides 1 and 2 and Gm apolipophoricin, were not described in *G*. *mellonella* previously. Another study, allowed the detection of a rich array of known or putative *G*. *mellonella* antimicrobial peptides or proteins in immune hemolymph namely, lysozyme, moricin-like peptides, cecropins, gloverin, Gm-proline rich peptide 1, Gm-proline rich peptide 2, Gm anionic peptide 1, Gm anionic peptide 2, galiomicin, gallerimycin, inducible serine protease inhibitor 2, 6tox and heliocin-like peptide [38]. Antibacterial activity has been observed in cell free hemolymphs (CFH) of *G*. *mellonella* challenged with *P*. *aeruginosa* [39] and it was found that antibacterial activity of CFH was noted at 9h after challenge, reaching a peak at 18h while traces were found at 30h post challenge. However, there is so far a lack of data using microorganisms with health promoting properties in the wax moth model host. In this context, only two studies have been recently published to our knowledge. The first study [21] demonstrates that cells and culture supernatants of *L*. *acidophilus* ATCC 4356 can serve as prophylactic or therapeutic agents against candidiasis in *G*. *mellonella* by reducing the number of yeast cells in the larval hemolymph and increasing the survival of infected larvae. In the second study [22], in the same model host, it was shown that eugenol and treatment with LAB, either alone or in combination, enhanced the survival rates of *G*. *mellonella* larvae infected with the pathogen *L*. *monocytogenes* and significantly reduced the pathogen’s adhesion and invasion to intestinal cells while also hemolysin production, E-cadherin binding and virulence gene expression was significantly decreased.

The objective of the present study was to evaluate the effect of two selected LAB strains originating from black olive fermentation namely, *L*. *pentosus* B281 and *L*. *plantarum* B282 [40] against the pathogenic bacteria *L*. *monocytogenes* and *S*. *aureus* using the *G*. *mellonella* infection model. The selected LAB strains have been used in the present study because of their previously shown functional and technological properties. Strain B282 has been demonstrated to possess 9 genes belonging to *pln* locus and involved in plantaricin production [41]. However, no relevant information is available for the other LAB strains. Their probiotic potential has been further assessed by *in vitro* tests [13] while their adhesion and anti-proliferative properties in cancer cell lines have been recently reported [42]. Moreover, their use as starter cultures in olive fermentation and survival in high numbers on the final product has been reported [43,44].

## Materials and Methods

### Microorganisms tested and preparation of inoculum

The LAB strains *L*. *pentosus* B281, *L*. *plantarum* B282 and *L*. *rhamnosus* GG were employed in the present study. The former two strains (B281 and B282) belong to the culture collection of the Laboratory of Microbiology and Biotechnology of Foods (FMCC) of the Agricultural University of Athens, whereas the latter strain (LGG) belongs to the culture collection of the Laboratory of Dairy Research (ACA-DC) of the same University, and was kindly provided by Prof. E. Tsakalidou. The pathogens *S*. *aureus* B134 (ATCC 6538) and *L*. *monocytogenes* B129 were also taken from the FMCC collection. The former microorganism was isolated from human lesion and the latter from ready-to-eat frozen meal. All microorganisms were maintained as stock cultures in de Man- Rogosa–Sharpe broth (MRS, Lab M, Heywood, UK) for LAB strains and Tryptic Soy broth (TSB, Lab M, Heywood, UK) for pathogenic strains, supplemented with 20% glycerol and stored at -80°C. LAB strains were revived by adding 10 μL of the stock culture in 10 mL MRS broth and incubating at 30°C for 24 h. Working cultures were obtained by adding 100 μL of the revived culture in 10 mL MRS and incubating at 30°C for 24h. Pathogenic strains were revived by adding 10 μL of the stock culture in 10 mL TSB and incubating at 37°C for 24h. Working cultures were obtained by adding 100 μL of the revived culture in 10 mL TSB and incubating at 37°C for 16–18 h. All working cultures were centrifuged at 5,000 g for 10 min at 4°C and the resulting pellet was resuspended in 1× Phosphate Buffer Saline (PBS, Oxoid) resulting in a final concentration of *ca*. 9.0 log CFU/mL as assessed by plate counting. Serial decimal dilutions were further prepared in the same PBS medium to adjust the desired concentration of bacterial cells.

### *Galleria mellonella* treatments

The effect of LAB upon the course of infection by the pathogens *S*. *aureus* B134 and *L*. *monocytogenes* B129 was assessed by two main series of experiments namely, prior to infection (within the context of prolepsis/prophylaxis) and post infection (within the context of therapy). Within the context of prophylaxis, larvae were challenged with viable or heat-killed cells of each LAB strain at the dose of 10^4^ CFU/larva. In preliminary trials, high initial levels of LAB (ranging from 10^5^ to 10^7^ CFU/larvae) were administered and their virulence potential on the larvae was monitored. It was observed that high initial populations of LAB resulted in high mortality of the larvae depending on the injected dose. Specifically, for LAB strains *L*. *pentosus* B281 and *L*. *plantarum* B282 the infectious dose was 5.5 log CFU/larva, whereas for *L*. *rhamnosus* GG the infectious dose was 6.0 log CFU/larva. To tackle this effect, it was decided to lower the inoculated LAB dose to 10^4^ CFU/larvae, a dose that did not affect the survival of the larvae. Infection with the target pathogen took place at selected time points post injection with LAB namely, 6 and 24 h, at an infection dose of 10^6^ CFU/larva. For the heat killing treatment, LAB cultures were placed in a water bath at 60°C for 30 min. The efficacy of the heat killing was assessed by culture.

Within the context of therapy, larvae were infected with 10^6^ CFU/larva of the target pathogen and subsequently treated with 1/10 cell free supernatants of LAB cultures. In order to obtain cell-free supernatants, the LAB cultures were centrifuged at 5000 g for 10 min at 4°C and then the supernatant was filter sterilized using 0.25 μm filter.

### *Galleria mellonella* injections

Final instar larvae of the greater wax moth *Galleria mellonella*, weighing 250–350 mg, were purchased from a local vendor (Reptilia Nostra, Pet Servive, Athens, Greece), stored at 15°C in the dark and used within 7 days. Prior to the different injection treatments, the larvae bodies were rinsed with 70% alcohol.

For the prophylaxis experiments, 10 μL inoculum containing 10^4^ CFU viable or heat-killed cells of each LAB strain were directly injected into larval hemocoel using a Hamilton syringe via the last left proleg. Larvae were incubated at 37°C in the dark and at 6 or 24 h of incubation they were again injected via the last right proleg with 10 μL inoculum containing 10^6^ CFU of the pathogen. Then, the larvae were placed again at 37°C in the dark. The experiment was repeated twice for each pathogen at independent time-points. Appropriate control groups were also included namely, one group receiving PBS injections for general survival, one group receiving injection with the pathogen alone, and two groups receiving PBS injection at 6 and 24 h prior to infection with the pathogens (non-LAB control groups). Results from all assays were compared with the non-LAB control groups that received PBS injections prior to infection.

In the case of experiments within the context of therapy, larvae were injected with a 10 μL inoculum containing 10^6^ CFU of target pathogen via the last left proleg and subsequently they were injected with 10 μL inoculum containing 1/10 diluted cell free supernatants of each LAB strain via the last right proleg. Then, the larvae were incubated at 37°C in the dark. The experiment was repeated twice for each pathogen at independent time-points. One group receiving PBS injection, one group receiving injection with the pathogen and one group receiving injection with the pathogen and then with PBS were included as control groups. Results from this experiment were compared with the control group that received injection with the pathogen and then received sham injections with PBS.

### Killing assays and microbiological analysis

For the killing assays, 16 randomly chosen larvae were used per treatment and the number of dead larvae was scored daily for a period of 7 days. Larvae were considered dead when they displayed no movement in response to touch. For all killing assays, Kaplan-Meier survival analysis was performed to determine the percentage of survival of infected larvae using STATA 6 and a *p*-value <0.05 was considered to be statistically significant in comparison with the control group. Specifically, the number of dead larvae was recorded each day and at the end of the assay the number of alive larvae was scored as censored. The persistence of the pathogen in the hemolymph was assessed by microbiological analysis at specific time intervals within 48 h after infection. Specifically, at 8, 16, 24 and 48 h post infection the hemolymph of 5 infected larvae was collected in a 2 mL pre-chilled eppendorf tube. In order to ensure sufficient number of live individuals in each time point to perform the analysis, a total of 30–35 larvae were used for each treatment. Once the hemolymph was collected, decimal serial dilutions with PBS were prepared and 0.1 mL of the appropriated dilution was spread on agar media to quantify the population of the pathogen. *L*. *monocytogenes* was enumerated on Listeria PALCAM agar base (Biolife, Milan, Italy) incubated at 37°C for 48 h, whereas *S*. *aureus* was counted on Baird Parker agar (Biolife, Milan, Italy) incubated at 37°C for 48 h. Results were expressed as log values of colony forming units per mL (log CFU/mL) of hemolymph. Microbiological data for each sampling point were subjected to one-way analysis of variance using the software JMP version 8.0 and significant differences (*p*- value ≤ 0.05) were determined by *t* test for paired samples.

## Results

### Effect of challenge with live and heat killed *Lactobacillus* cells on *L*. *monocytogenes* infection

**Fig 1:**
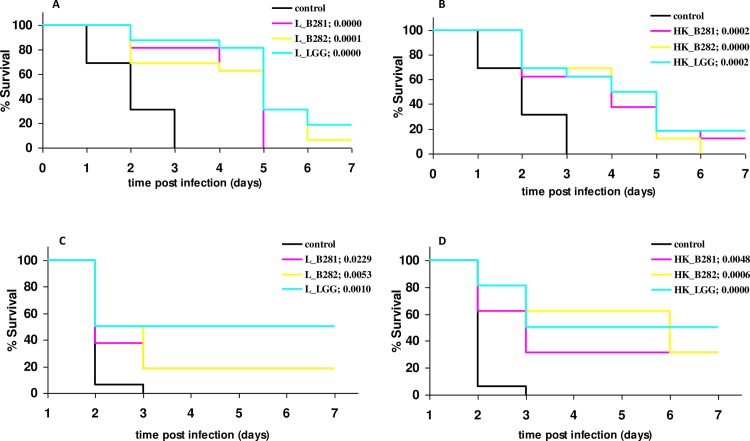
Kaplan-Meier survival plots of *G*. *mellonella* larvae by *L*. *monocytogenes* infection. Larvae were previously administered with 10^4^ live (**A, C**) and heat-killed (**B, D**) cells of *L*. *pentosus* B281(L_B281), *L*. *plantarum* B282 (L_B282) and *L*. *rhamnosus* LGG (L_LGG). LAB administration was performed at 6 (**A, B**) and 24 h (**C, D**) prior to infection. All groups from each treatment were compared with the control group using the log rank test. The corresponding *p*-values are given in the parenthesis for each group. Groups of 16 larvae were used for the killing assays.

The challenge with live or heat-killed cells of selected LAB strains of *G*. *mellonella* larvae as means to control *L*. *monocytogenes* infection was evaluated at 6 and 24 h prior to infection. Killing assays for the different treatments are presented in Fig 1. In the case of infection at 6 h post injection with LAB, individuals of the control group (group that received PBS injection prior to infection) started dying from the first day of infection and they all died by day 3 (Fig 1). Soon after the injection with the pathogen, melanization of the larvae was observed that became more intense with the progress of infection. Challenge with live (L) LAB cells resulted in slower killing that extended into day 4 or 5, depending on the LAB strain, with *p*-values < 0.0001 for groups L_B281, L_B282, and L_LGG (Fig 1A). The persistence of live LAB cells in the hemolymph is presented as supplementary file (S1 Fig). Overall, the population of all LAB strains was recovered in reduced populations at 8 h post infection and thereafter they were either stable or increased throughout the course of infection. Slower killing of infected larvae was noticeable as well in groups that received heat killed (HK) LAB cells, compared to the control group, with *p*-values 0.0002, 0.0000 and 0.0002 for groups HK_B281, HK_B282, and HK_LGG, respectively (Fig 1B). A different pattern was observed when infection took place at 24 h post challenge. In the control group, all larvae died within 3 days after infection with 15/16 (93.75%) of them dead at day 2. From day 3 onwards, administration with live LAB cells resulted in survival rates 18.75% for groups L_B281 (*p* 0.0229) and L_B282 (*p* 0.0053), and 50% for group L_LGG (*p* 0.0010) (Fig 1C). Further, injections with heat-killed LAB cells resulted in higher survival rates for groups HK_B281 and HK_B282 (31.25%) and *p*-values 0.0048 and 0.0006, respectively, while the same percentage of survival for group HK_LGG was observed (50%) with a *p*-value 0.0000 (Fig 1D).

**Fig 2:**
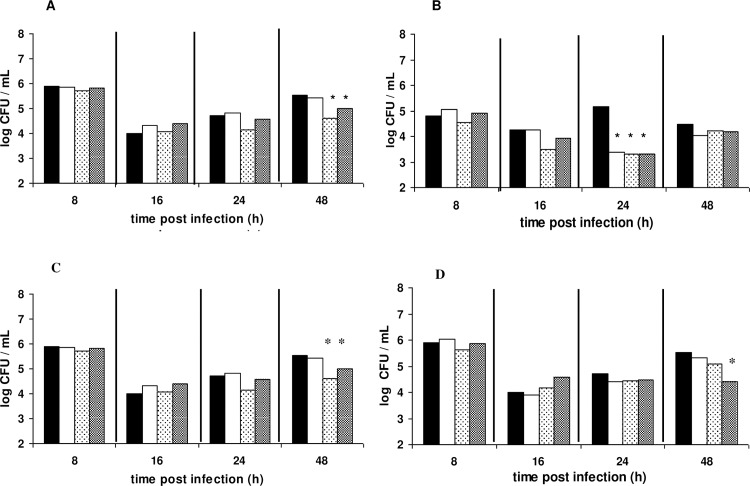
Population dynamics of *L*. *monocytogenes* in the host’s hemolymph. Larvae were injected with live (A,C) or heat-killed (B,D) cells of *L*. *pentosus* B281 (white bars), *L*. *plantarum* B282 (dotted bars) and *L*. *rhamnosus* LGG (grey bars). Infection with the pathogen took place at 6 (A, B) and 24 h (C, D) post LAB administration. Black bars correspond to the control group. Bars with asterisks show groups with significant differences in comparison with the control group with a *p*-value ≤ 0.05. A total of 30–35 larvae were used for each treatment in order to determine the population of the pathogen in the hemolymph.

The survival of the pathogen in the hemolymph was determined by microbiological analysis at specific time intervals within 48 h after infection. In the case of 6 h challenge (Fig 2A), the population (CFU) of the pathogen was recovered under all treatments at levels similar to the control group, with the exception of the 24 h time point where the pathogen significantly decreased in all immunized hemolymphs by 0.76–1.80 log units. Among live injected LAB strains, the highest log reduction was observed by strain B282 (1.26 log units, *p* 0.03104) in comparison with the control group, while all LAB strains administered as heat killed resulted in 1.80 log units reduction with *p*-values 0.0322, 0.0325 and 0.0356 for strains B281, B282, and LGG, respectively. In the case of 24 h challenge on the other hand, only live cells of B282 strain and heat killed cells of the reference LGG strain resulted in significant decrease of the pathogen at 48 h post infection with *p*-values 0.0279 and 0.0202, respectively (Fig 2B). All the observed significant differences as determined by *t* test for paired samples for the different sampling points are presented in Table 1.

**Table 1:** Significant differences as determined by *t* test for paired samples in the case of *Listeria monocytogenes* infection.

treatment	**6 h challenge with live LAB cells prior to infection**		
Time post infection (h)	Levels of compared pairs	Difference in compared counts	*p* value
24	Control^a^ - B282	1.258791	0.0104
	Control—LGG	1.007226	0.0219
	Control—B281	0.760553	0.0513
	B281—B282	0.498238	0.1458
	LGG—B282	0.251565	0.4142
	B281—LGG	0.246673	0.4226
treatment	**6 h challenge with heat killed LAB cells prior to infection**		
Time post infection (h)	Levels of compared pairs	Difference in compared counts	*p* value
24	Control^a^- LGG	1.859587	0.0322
	Control- B282	1.854983	0.0325
	Control- B281	1.799849	0.0356
	B281- LGG	0.059738	0.9226
	B281- B282	0.055134	0.9285
	B282- LGG	0.004604	0.9940
treatment	**24 h challenge with live LAB cells prior to infection**		
Time post infection (h)	Levels of compared pairs	Difference in compared counts	*p* value
48	Control^b^- B282	0.9458303	0.0279
	Control—LGG	0.8236724	0.0424
	B281- B282	0.5566137	0.1179
	B281- LGG	0.4344558	0.1960
	LGG—B282	0.3892166	0.2372
	Control- B281	0.1221578	0.6854
treatment	**24 h challenge with heat killed LAB cells prior to infection**		
Time post infection (h)	Levels of compared pairs	Difference in compared counts	*p* value
48	Control^b^ - LGG	1.105562	0.0202
	B281—LGG	0.878525	0.0411
	B282—LGG	0.652186	0.0921
	Control—B282	0.453377	0.2001
	Control—B281	0.227037	0.4855
	B281—B282	0.226339	0.4868
treatment	**Infection with *Listeria monocytogenes* and subsequent treatment with LAB supernatants**		
Time post infection (h)	Levels of compared pairs	Difference in compared counts	*p* value
24	Control^c^ - LGG	2.239195	0.0005
	Control–B282	1.979909	0.0009
	Control—B281	1.560265	0.0022
	B281—LGG	0.678930	0.0376
	B281 –B282	0.419644	0.1315
	B282—LGG	0.259286	0.3074
48	Control^c^ - B281	1.444702	< .0001
	Control–B282	1.440526	< .0001
	Control–LGG	1.212847	0.0002
	LGG–B281	0.231855	0.0565
	LGG–B282	0.227679	0.0594
	B282—B281	0.004176	0.9641

^a^: Refers to the groups receiving PBS injection at 6 h prior to infection.

^b^: Refers to the groups receiving PBS injection at 24 h prior to infection.

^c^: Refers to the groups receiving PBS injection right after the infection.

### Effect of challenge with viable and heat killed *Lactobacillus* cells on *S*. *aureus* infection

**Fig 3:**
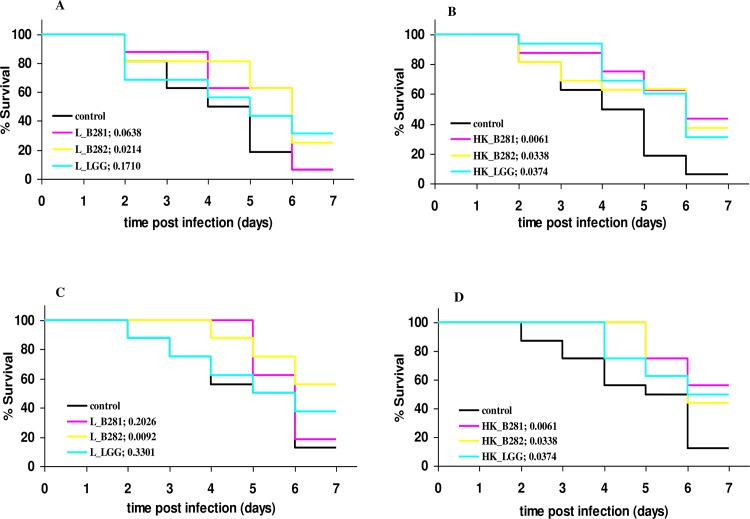
Kaplan-Meier survival plots of *G*. *mellonella* larvae by *S*. *aureus* infection. Larvae were previously administered with 10^4^ live (**A, C**) and heat-killed (**B, D**) cells of *L*. *pentosus* B281(L_B281), *L*. *plantarum* B282 (L_B282) and *L*. *rhamnosus* LGG (L_LGG). LAB administration was performed at 6 (**A, B**) and 24 h (**C, D**) prior to infection. All groups from each treatment were compared with the control group using the log rank test. The corresponding *p*-values are given in the parenthesis for each group. Groups of 16 larvae were used for the killing assays.

The survival plots of *G*. *mellonella* larvae against *S*. *aureus* for the different treatments are illustrated in Fig 3. In comparison to *L*. *monocytogenes*, injections with *S*. *aureus* in non-treated larvae did not result in fast killing of infected individuals but rather in an attenuated infection. Melanization of the larvae was also observed in the case of *S*. *aureus* infection. However, in the case of *L*. *monocytogenes*, the phenomenon appeared to be more immediate, since it started within minutes after infection compared to *S*. *aureus* that started at approximately 1.5 h after infection. In both cases however, dead individuals appeared to be completely black. However, higher survival rates were obtained in treated larvae, in both challenge times, with the highest observed in treatments with heat-killed cells of all tested LAB strains.

**Fig 4:**
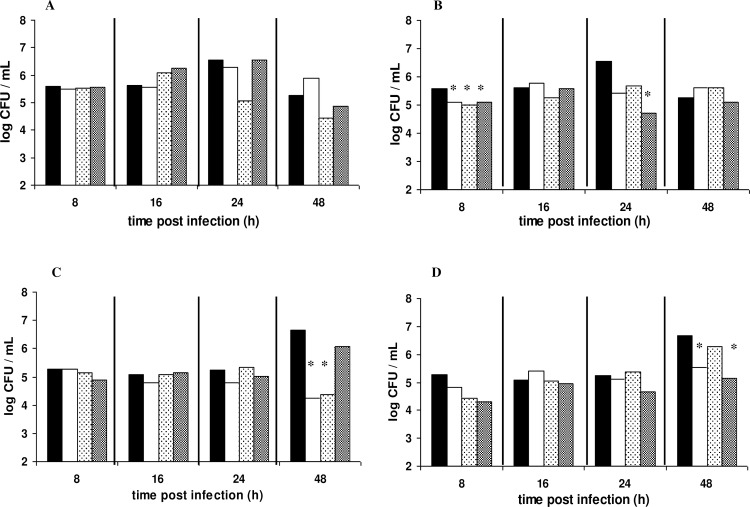
Population dynamics of *S*. *aureus* in the host’s hemolymph. Larvae were injected with live (A,C) or heat-killed (B,D) cells of *L*. *pentosus* B281 (white bars), *L*. *plantarum* B282 (dotted bars) and *L*. *rhamnosus* LGG (grey bars). Infection with the pathogen took place at 6 (A, B) and 24 h (C, D) post LAB administration. Black bars correspond to the control group. Bars with asterisks show groups with significant differences in comparison with the control group with a *p*-value ≤ 0.05. A total of 30–35 larvae were used for each treatment in order to determine the population of the pathogen in the hemolymph.

The persistence of *S*. *aureus* in treated and non-treated hemolymph is shown in Fig 4. In the 6 h challenged group, the pathogen in the control group was recovered at 5.6 ± 0.19 log CFU/mL at 8 h post infection reaching a peak of 6.5 ± 0.7 log CFU/mL at 24 h and dropping again at 5.3 ± 0.3 log CFU/mL at 48 h post infection. Overall, the same trend was observed in groups that received live LAB cells with the exception of the group that received B282, where the pathogen was reduced at 24 and 48 h post infection. The persistence of live LAB cells in the host’s hemolymph is illustrated in supplementary S2 Fig. It was observed that LAB population in the hemolymph presented a decrease at 8h post infection followed by an increase thereafter depending on the strain and type of treatment. Only in the case of 24 h challenged group, the population of LGG did not follow this trend and remained unchanged throughout infection. Significant differences for the pathogen’s persistence in the hemolymph are summarized in Table 2. In the case of administration with heat-killed LAB cells, a small but significant decrease of the pathogen in the early stage of infection (8 h) for all strains was observed with *p*-values 0.0096, 0.0161 and 0.0175 for strains B281, B282, and LGG, respectively. A noticeable decrease at 24 h for strains B281 and LGG was observed, that was statistically significant only for the latter strain (*p* 0.0240). For groups that received LAB 24 h prior to infection, the pathogen in non- treated hemolymphs remained unchanged until 24 h post infection at around 5.0 log CFU/mL and reached a peak of 6.7 log CFU/mL at 48 h post infection. A significant decrease was observed at this time point in groups that received live cells of B281 (*p* 0.0099) and B282 (*p* 0.0122) strain as well as in groups that received heat killed cells of B281 (*p* 0.0399) and LGG (*p* 0.0150) strain.

**Table 2:** Significant differences as determined by *t* test for paired samples in the case of *Staphylococcus aureus* infection.

treatment	**6 h challenge with live LAB cells prior to infection**		
Time post infection (h)	Levels of compared pairs	Difference in compared counts	*p* value
48	B281—B282	1.432651	0.0349
	B281—LGG	1.028228	0.0873
	Control^a^ - B282	0.823909	0.1453
	B281—Control	0.608742	0.2531
	Control—LGG	0.419486	0.4100
	LGG—B282	0.404422	0.4255
treatment	**6 h challenge with heat killed LAB cells prior to infection**		
Time post infection (h)	Levels of compared pairs	Difference in compared counts	*p* value
8	Control^a^ - B282	0.5790898	0.0096
	Control—B281	0.4973566	0.0161
	Control—LGG	0.4853397	0.0175
	LGG—B282	0.0937501	0.4929
	281—B282	0.0817332	0.5469
	LGG—B281	0.0120169	0.9277
24	Control^a^ - LGG	1.842666	0.0240
	Control—B281	1.114637	0.0988
	B282—LGG	0.989006	0.1301
	Control—B282	0.853659	0.1762
	B281—LGG	0.728028	0.2343
	B282—B281	0.260978	0.6423
treatment	**24 h challenge with live LAB cells prior to infection**		
Time post infection (h)	Levels of compared pairs	Difference in compared counts	*p* value
48	Control^b^ - B281	2.411390	0.0099
	Control—B282	2.266589	0.0122
	LGG—B281	1.839383	0.0244
	LGG—B282	1.694583	0.0315
	Control—LGG	0.572006	0.3349
	B282—B281	0.144800	0.7953
treatment	**24 h challenge with heat killed LAB cells prior to infection**		
Time post infection (h)	Levels of compared pairs	Difference in compared counts	*p* value
8	Control^b^ - LGG	0.9751013	0.0497
	Control—B282	0.8406786	0.0744
	B281—LGG	0.5258059	0.2078
	Control—B81	0.4492953	0.2690
	B281—B282	0.3913833	0.3265
	B282—LGG	0.1344227	0.7207
48	Control^b^ - LGG	1.517779	0.0150
	B282—LGG	1.132904	0.0379
	Control—B281	1.113198	0.0399
	B282—B281	0.728323	0.1211
	B281—LGG	0.404581	0.3368
	Control—B282	0.384875	0.3582

^a^: Refers to the groups receiving PBS injection at 6 h prior to infection.

^b^: Refers to the groups receiving PBS injection at 24 h prior to infection.

### Treatment of *L*. *monocytogenes* and *S*. *aureus* infection with *Lactobacillus* cell-free supernatants (CFSs)

**Fig 5:**
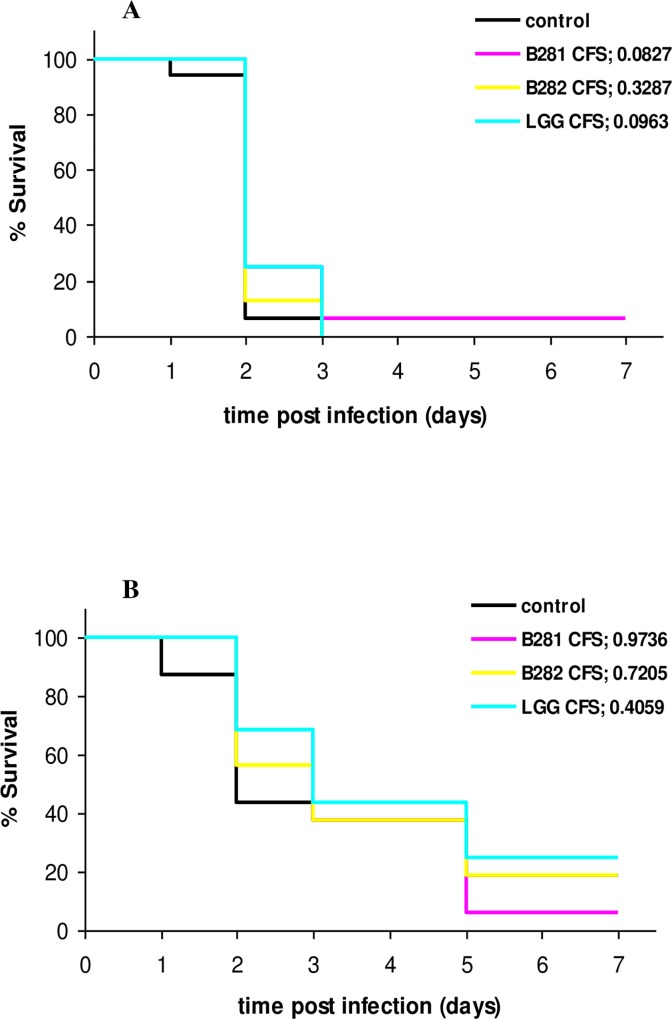
Killing of *G*. *mellonella* by *L*. *monocytogenes* and *S*. *aureus* after treatment with LAB CFS. Larvae were infected (A) with *L*. *monocytogenes* and subsequently treated with 1/10 CFS of cultures of *L*. *pentosus* B281, *L*. *plantarum* B282 and *L*. *rhamnosus* LGG (LGG cfs; *p* 0.0963) and (B) with *S*. *aureus* and subsequently treated with 1/10 CFS of cultures of *L*. *pentosus* B281, *L*. *plantarum* B282 and *L*. *rhamnosus* LGG. The corresponding *p*-values are given in the legend for each group. Groups of 16 larvae were used for the killing assays.

**Fig 6:**
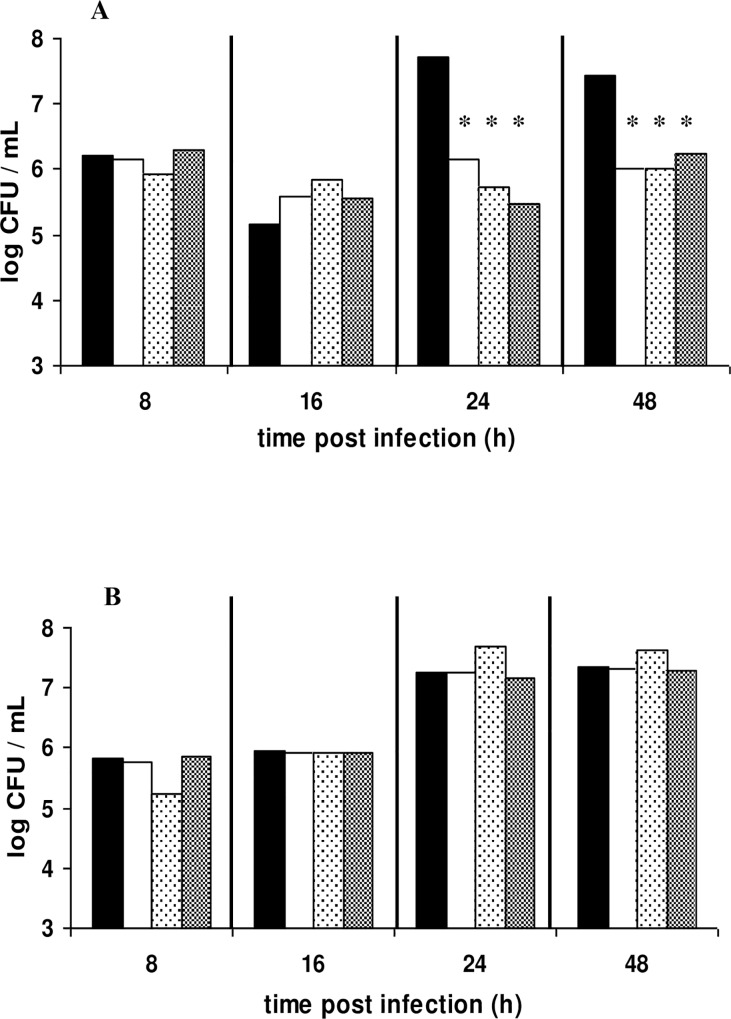
Population dynamics of *L*. *monocytogenes* and *S*. *aureus* in *G*. *mellonella* hemolymph after treatment with LAB CFS. Larvae were infected with (A) *L*. *monocytogenes* and (B) *S*. *aureus* and susequently treated with 1/10 CFS of cultures of B281, B282 and LGG LAB strains. Bars with asterisks show groups with significant differences in comparison with the control group with a *p*-value ≤ 0.05. A total of 30–35 larvae were used for each treatment in order to determine the population of the pathogen in the hemolymph.

Cell free supernatants from LAB cultures were tested as treatment therapy in *G*. *mellonella* larvae infected with *L*. *monocytogenes* and *S*. *aureus*. For both pathogens, no prolonged survival was observed in any of the groups treated with CFSs in comparison with the control cases (Fig 5). However, at 24 and 48 h post infection with *L*. *monocytogenes*, the pathogen was found significantly reduced by 1.2–2.0 log units and *p*-values ≤ 0.05 (Fig 6A, Table 1). Microbiological data for *S*. *aureus* infection indicated that CFS treatments had no effect in the host’s defense against infection (Fig 6B).

## Discussion

One of the aspects of the present work was to investigate the protective potential of selected LAB strains, against the pathogens *L*. *monocytogenes* and *S*. *aureus*. A significant attenuation was observed in *Listeria* infection using both live and heat-killed cells of LAB strains, depending on the strain and challenge period prior to infection (Figs 1 and 2). In the case of *S*. *aureus* infection, live cells of B282 strain attenuated infection under both challenge periods while heat-killed cells of all strains attenuated infection under 24 h challenge period (Figs 3 and 4). The *G*. *mellonella* infection model has been previously reported for its efficacy in studying *Listeria* pathogenesis while the larval hemolymph has been further proposed as a source for anti-listeria therapeutics due to increased expression of defense peptides after infection [29,30]. The virulence of *S*. *aureus* in *G*. *mellonella* was first assessed by Peleg et al. [45] while later studies support the use of the wax moth for testing antistaphylococcal agents [28,46,47]. In one of these studies [46], larvae of the wax moth model were efficiently used as an invertebrate model for assessing virulence of SCV (Small Colony Variant)-like *S*. *aureus* using the same *S*. *aureus* strain as in the present study. However, no direct comparison can be discussed with the results of the present work since, the scope between these experiments are different and to the best of our knowledge there are no studies in the literature assessing the efficacy of LAB administration against this pathogen. A recently published work [21], investigated the effect of live cells supernatant filtrates of *L*. *acidophilus* ATCC 4356 at 1 h pre and post infection with *C*. *albicans* in *G*. *mellonella* larvae and it was observed that treatments with the LAB culture or filtrate resulted in significantly higher survival in comparison with the control group.

The definition given by FAO/WHO [1] for probiotics implies that intake of probiotics as live microorganisms is a prerequisite in order to exert a health benefit on the host. This definition however neglects the role of heat-killed cells as elicitors of immune responses. For this reason, heat-killed LAB cells were also used in the present study as potential protective factors against infection. A number of published studies provide evidence that the use of heat-killed LABs poses a series of positive health effects in mouse animal models. Heat-killed cells of *L*. *casei* strain YIT9018 species have been previously reported for their antimetastatic properties in mouse tumor systems [48]. *L*. *casei* strain Shirota [49], *L*. *brevis* SBC8803 [50] and a number of other LAB strains [51] have been reported for their preventive or ameliorating potential allergic diseases in ovalbumin (OA)-sensitized mouse spleenocytes by reduction of immunoglobulin E (IgE) and improvement of the balance between T helper cells type 1 (Th1) and type 2 (Th2). In addition, heat-killed strains of *L*. *acidophilus*, *L*. *plantarum*, *Lactobacillus casei*, *L*. *pentosus* have also been documented in the literature for their efficacy against *Salmonella* Typhimurium [52,53], *Streptococcus pneumoniae* [54,55] and influenza virus [56,57] invasions. To our knowledge, the use of heat-killed cells of microorganisms with probiotic potential in the *G*. *mellonella* model system has not been reported in the literature. The prolonged survival which was observed at both challenge periods for both target pathogens further highlight that heat killing of selected bacterial strains can be promising against infection.

A number of studies published in the literature demonstrate positive attributes of lactobacilli metabolic by-products. Growth inhibition of pathogens due to CFS from *L*. *rhamnosus*, *L*. *acidophilus* and *B*. *lactis* has been previously reported [58,59,60]. In this sense, in the present study, the efficacy of LAB CFS as therapeutic agents against *L*. *monocytogenes* and *S*. *aureus* infection was further assessed in larvae of the wax moth. However, no prolonged survival of infected individuals was observed for both pathogens. Only in the case of *L*. *monocytogenes* infection, the pathogen was recovered at significantly lower numbers at 24 and 48 h post infection. In the study of Vilela et al. [21], CFS from *L*. *acidophilus* ATCC 4356 significantly prolonged *G*. *mellonella* survival against *C*. *albicans* infection with approximately 20% of the larvae being still alive by the end of the experiment while this survival prolongation was observed when it was administered both as prophylactic and therapeutic agent.

## Conclusion

The results of the present study provide evidence that *L*. *pentosus* B281 and *L*. *plantarum* B282 have the potential of antagonizing the important pathogens *L*. *monocytogenes* and *S*.*aureus* under *in vivo* conditions. The degree to which each strain exerts this property highly depends on the type of cells as well as the challenge time. The use of heat-killed LAB cells effectively decreased the pathogen burden in the larval hemolymph while short challenge periods prior to infection are recommended. Moreover, the use of CFSs of the specific bacterial strains does not seem to pose significant effect to pathogenesis, at least in the concept of therapy. Overall, the results of the present work can further assist in the broader use of the specific host as a low cost *in vivo* tool to better understand interactions between beneficial and pathogenic microorganisms. In addition, since research on the probiotic potential of microorganisms originating from olives is still recent, the use of the specific host can serve as a preliminary step for high throughput experiments prior to the use of murine models and clinical studies.

## Supporting Information

S1 Fig Persistence of injected LAB in the hemolymph after infection with *L*. *monocytogenes*. Larvae were injected with *L*. *pentosus* B281 (black bars), *L*. *plantarum* B282 (white bars) and *L*. *rhamnosus* GG (grey bars) at 6h (A) and 24h (B) prior to infection with the pathogen.
(TIF)

S2 Fig Persistence of injected LAB in the hemolymph after infection with *S*. *aureus*. Larvae were injected with *L*. *pentosus* B281 (black bars), *L*. *plantarum* B282 (white bars) and *L*. *rhamnosus* GG (grey bars) at 6h (A) and 24h (B) prior to infection with the pathogen.
(TIF)

## References

[1] FAO/WHO. Probiotics in food. Health and nutritional properties and guidelines for evaluation. Report of a joint FAO/WHO working group on drafting guidelines for the evaluation of probiotics in food. London, Ontario, Canada, 30 April-1 May 2002.

[2] Salminen S, Ouwehand AC, Isolauri E. Clinical applications of probiotic bacteria. Int Dairy J. 1998; 8: 563–572.

[3] Wollowski I, Rechkemmer G, Pool-Zobel BL. Protective role of probiotics and prebiotics in colon cancer. Am J Clin Nutr. 2001; 73: 451S–455S. 11157356 10.1093/ajcn/73.2.451s

[4] Cross ML. Microbes versus microbes: immune signals generated by probiotic lactobacilli and their role in protection against microbial pathogens. FEMS Immunol Med Microbiol. 2002; 34: 245–253. 12443824 10.1111/j.1574-695X.2002.tb00632.x

[5] Commane D, Hughes R, Shortt C, Rowland I. The potential mechanisms involved in the anti-carcinogenic action of probiotics. Mutat Res. 2005; 591(1–2): 276–289. 16095630 10.1016/j.mrfmmm.2005.02.027

[6] Tang MLK, Lahtinen E, Boyle RJ. Probiotics and prebiotics: clinical effects in allergic disease. Curr Opin Pediatr. 2010; 22: 626–634. 10.1097/MOP.0b013e32833d9728 20733491

[7] Gupta S, Abu-Ghannam N. Probiotic fermentation of plant based products: possibilities and opportunities. Crit Rev Food Sci Nutr. 2012; 52: 183–199. 10.1080/10408398.2010.499779 22059963

[8] Lavermicocca P, Valerio F, Lonigro SL, De Angelis M, Morelli L, Callegari ML, et al Study of adhesion and survival of lactobacilli and bifidobacteria on table olives with the aim of formulating a new probiotic food. Appl Environ Microbiol. 2005; 71: 4233–4240. 16085808 10.1128/AEM.71.8.4233-4240.2005 PMC1183302

[9] De Bellis P, Valerio F, Sisto A, Lonigro SL, Lavermicocca P. Probiotic table olives: Microbial populations adhering on olive surface in fermentation sets inoculated with the probiotic strain *Lactobacillus paracasei* IMPC2.1 in an industrial plant. Int J Food Microbiol. 2010; 140: 6–13. 10.1016/j.ijfoodmicro.2010.02.024 20226556

[10] Bevilacqua A, Altieri C, Corbo MR, Sinigaglia M, Ouoba LI. Characterization of lactic acid bacteria isolated from Italian Bella di Cerignola table olives: selection of potential multifunctional starter cultures. J Food Sci. 2010; 75: M536–M544. 10.1111/j.1750-3841.2010.01793.x 21535510

[11] Abriouel H, Benomar N, Cobo A, Caballero N, Fernández Fuentes MÁ, Pérez-Pulido R, et al Characterization of lactic acid bacteria from naturally-fermented Manzanilla Aloreña green table olives. Food Microbiol. 2012; 32: 308–316. 10.1016/j.fm.2012.07.006 22986194

[12] Peres CM, Peres C, Hernández-Mendoza A, Xavier Malcata F. Review on fermented plant materials as carriers and sources of potentially probiotic lactic acid bacteria–with an emphasis on table olives. Trends in Food Sci Technol. 2012; 26: 31–42.

[13] Argyri AA, Zoumpopoulou G, Karatzas K-AG, Tsakalidou E, Nychas G-JE, Panagou EZ, et al Selection of potential probiotic lactic acid bacteria from fermented olives by *in vitro* tests. Food Microbiol. 2013; 33: 282–291. 10.1016/j.fm.2012.10.005 23200662

[14] Bautista-Gallego J, Arroyo-López FN, Rantsiou K, Jiménez-Díaz R, Garrido-Fernández A, Cocolin L. Screening of lactic acid bacteria isolated from fermented table olives with probiotic potential. Food Res Int. 2013; 50: 135–142.

[15] Botta C, Langerholc T, Cencič A, Cocolin L. *In vitro* selection and characterization of new probiotic candidates from table olive microbiota. PLoS One. 2014; 9 (4): e99457. 24714329 10.1371/journal.pone.0094457 PMC3979845

[16] Glavis-Bloom J, Muhammed M, Mylonakis E. Of model hosts and man: Using *Caenorhabditis elegans*, *Drosophila melanogaster* and *Galleria mellonella* as model hosts for infectious disease research In: Mylonakis E, Ausubel FM, Gilmore M, Casadevall A, editors. Recent Advances on Model Hosts, vol. 710 Advances in Experimental Medicine and Biolgy Springer New York; 2012 pp. 11–17. 10.1007/978-1-4419-5638-5_2 22127881

[17] Lee J, Yun HS, Cho KW, Oh S, Kim SH, Chun T, et al Evaluation of probiotic characteristics of newly isolated *Lactobacillus* spp.: immune modulation and longevity. Int J Food Microbiol. 2011; 148: 80–86. 10.1016/j.ijfoodmicro.2011.05.003 21652104

[18] Komura T, Ikeda T, Yasui C, Saeki S, Nishikawa Y. Mechanism underlying prolongevity induced by bifidobacteria in *Caenorhabditis elegans*. Biogerontology. 2013; 14: 73–87. 10.1007/s10522-012-9411-6 23291976

[19] Kim Y, Mylonakis E. *Caenorhabditis elegans* immune conditioning with the probiotic bacterium *Lactobacillus acidophilus* strain NCFM enhances gram positive immune responses. Infec Immun. 2012; 80: 2500–2508. 22585961 10.1128/IAI.06350-11 PMC3416467

[20] Jones RM, Luo L, Ardita CS, Richardson AN, Kwon YM, Mercante JW, et al Symbiotic lactobacilli stimulate gut epithelial proliferation via Nox-mediated generation of reactive oxygen species. The EMBO J. 2013; 32: 3017–3028. 10.1038/emboj.2013.224 24141879 PMC3844951

[21] Vilela SF, Barbosa JO, Rossoni RD, Santos JD, Prata MC, Anbider AL, et al *Lactobacillus acidophilus* ATCC 4356 inhibits biofilm formation by *Candida albicans* and attenuates the experimental candidiasis in *Galleria mellonella*. Virulence. 2015; 6(1): 29–39. 10.4161/21505594.2014.981486 25654408 PMC4603435

[22] Upadhyay A, Upadhyaya I, Mooyottu S, Venkitanarayanan K. Eugenol in combination with lactic acid bacteria attenuate *Listeria monocytogenes* virulence *in vitro* and in invertebrate model, *Galleria mellonella*. J Med Microbiol 2016; 10.1099/jmm.0.000251 27002648

[23] Cotter G, Doyle S, Kavanagh K. Development of an insect model for the *in vivo* pathogenicity testing of yeasts. FEMS Immunol Med Microbiol. 2000; 27: 163–169. 10640612 10.1111/j.1574-695X.2000.tb01427.x

[24] Peleg AY, Jara S, Monga D, Eliopoulos GM, Moellering RC Jr, Mylonakis E. *Galleria mellonella* as a model system to study *Acinetobacter baumannii* pathogenesis and therapeutics. Antimicrob Agents Chemother. 2009; 53: 2605–2609. 10.1128/AAC.01533-08 19332683 PMC2687231

[25] Jander G, Rahme LG, Ausubel FM. Positive correlation between virulence of *Pseudomonas aeruginosa* mutants in mice and insects. J Bacteriol. 2000; 182: 3843–3845. 10851003 10.1128/jb.182.13.3843-3845.2000 PMC94559

[26] Miyata S, Casey M, Frank DW, Ausubel FM, Drenkard E. Use of the *Galleria mellonella* caterpillar as a model host to study the role of the type III secretion system in *Pseudomonas aeruginosa* pathogenesis. Infect Immun. 2003; 71: 2404–2413. 12704110 10.1128/IAI.71.5.2404-2413.2003 PMC153283

[27] Champion OL, Cooper IA, James SL, Ford D, Karlyshev A, Wren BW, et al *Galleria mellonella* as an alternative infection model for *Yersinia pseudotuberculosis*. Microbiology 2009; 155: 1516–1522. 10.1099/mic.0.026823-0 19383703

[28] Desbois AP, Coote PJ. Wax moth larva (*Galleria mellonella*): an *in vivo* model for assessing the efficacy of antistaphylococcal agents. J Antimicrob Chemother. 2011; 66: 1785–1790. 10.1093/jac/dkr198 21622972

[29] Mukherjee K, Altincicek B, Hain T, Domman E, Vilcinskas A, Chakraborty T. *Galleria mellonella* as a model system for studying *Listeria* pathogenesis. Appl Environ Microbiol. 2010; 76: 310–317. 10.1128/AEM.01301-09 19897755 PMC2798647

[30] Mukherjee K, Mraheil MA, Silva S, Müller D, Cemic F, Hemberger J, et al Anti-*Listeria* activities of *Galleria mellonella* hemolymph proteins. Appl Environ Microbiol. 2011; 77: 4237–4240. 10.1128/AEM.02435-10 21531838 PMC3131625

[31] Michaux C, Sanguinetti M, Reffuveille F, Auffray Y, Posteraro B, Gilmore MS, et al SlyA is a transcriptional regulator involved in the virulence of *Enterococcus faecalis*. Infect Immun. 2011; 79: 2638–2645. 10.1128/IAI.01132-10 21536798 PMC3191995

[32] Fuchs BB, Eby J, Nobile CJ, El Khoury JB, Mitchell AP, Mylonakis E. Role of filamentation in *Galleria mellonella* killing by *Candida albicans*. Microbes Infect. 2010; 12: 488–496. 10.1016/j.micinf.2010.03.001 20223293 PMC2883670

[33] Mylonakis E, Moreno R, El Khoury JB, Idnurm A, Heitman J, Calderwood SB, et al *Galleria mellonella* as a model system to study *Cryptococcus neoformans* pathogenesis. Infect Immun. 2005; 73: 3842–3850. 15972469 10.1128/IAI.73.7.3842-3850.2005 PMC1168598

[34] Bergin D, Murphy L, Keenan J, Clynes M, Kavanagh K. Pre-exposure to yeast protects larvae of *Galleria mellonella* from a subsequent lethal infection by *Candida albicans* and is mediated by the increased expression of antimicrobial peptides. Microbes Infect. 2006; 8: 2105–2112. 16782387 10.1016/j.micinf.2006.03.005

[35] Mowlds P, Barron A, Kavanagh K. Physical stress primes the immune response of *Galleria mellonella* larvae to infection by *Candida albicans*. Microbes Infect. 2008; 10: 628–634. 10.1016/j.micinf.2008.02.011 18457977

[36] Fallon JP, Troy N, Kavanagh K. Pre-exposure of *Galleria mellonella* larvae to different doses of *Aspergillus fumigatus* conidia causes differential activation of cellular and humoral immune responses. Virulence. 2011; 2 (5): 413–421. 10.4161/viru.2.5.17811 21921688

[37] Cytrynska M, Mak P, Zdybicka-Barabas A, Suder P, Jakubowicz T. Purification and characterization of eight peptides from Galleria mellonella immune hemolymph. Peptides. 2007; 28: 533–546. 17194500 10.1016/j.peptides.2006.11.010

[38] Brown SE, Howard A, Kasprzak AB, Gordon KH, East PD. A peptidomics study reveals the impressive antimicrobial peptide arsenal of the wax moth *Galleria mellonella*. Insect Biochem Molec. 2009; 39: 792–800. 10.1016/j.ibmb.2009.09.004 19786100

[39] Andrejko M, Mizerska-Dudka M, Jakubowicz T. Antibacterial activity *in vivo* and *in vitro* in the hemolymph of *Galleria mellonella* infected with *Pseudomonas aeruginosa*. Comp Biochem Physiol B. 2009; 152: 118–123. 10.1016/j.cbpb.2008.10.008 18996217

[40] Doulgeraki AI, Pramateftaki P, Argyri AA, Nychas G-JN, Tassou CC, Panagou EZ. Molecular characterization of lactic acid bacteria isolated from industrially fermented Greek table olives. LWT-Food Sci Technol. 2013; 50: 353–356.

[41] Doulgeraki AI, Paraskevopoulos N, Nychas G-JN, Panagou EZ. An in vitro study of *Lactobacillus plantarum* strains for the presence of plantaricin genes and their potential control of the table olive microbiota. A Van Leeuw. 2013; 103:821–832. 10.1007/s10482-012-9864-2 23224439

[42] Saxami G, Karapetsas A, Lamprianidou E, Kotsianidis I, Chlichlia A, Tassou C, et al Two potential probiotic lactobacillus strains isolated from olive microbiota exhibit adhesion and anti-proliferative effects in cancer cell lines. J Funct Foods. 2016; 24: 461–471.

[43] Blana VA, Grounta A, Tassou CC, Nychas G-JE, Panagou EZ. Inoculated fermentation of green olives with potential probiotic *Lactobacillus pentosus* and *Lactobacillus plantarum* starter cultures isolated from industrially fermented olives. Food Microbiol. 2014; 38: 208–218. 10.1016/j.fm.2013.09.007 24290645

[44] Grounta A, Doulgeraki AI, Nychas G-JE, Panagou EZ. Biofilm formation of Conservolea natural black olives during single and combined inoculation with a functional *Lactobacillus pentosus* starter culture. Food Microbiol. 2015; 56: 35–44. 10.1016/j.fm.2015.12.002 26919816

[45] Peleg AY, Monga D, Pillai S, Mylonakis E, Moellering RC Jr, Eliopoulos GM. Reduced susceptibility to vancomycin influences pathogenicity in *Staphylococcus aureus* infection. J Infect Dis. 2009; 199: 532–536. 10.1086/596511 19125671 PMC3750955

[46] Latimer J, Forbes S, McBain AJ. Attenuated virulence and biofilm formation in *Staphylococcus aureus* following sublethal exposure to triclosan. Antimicrob Agents Chemother. 2012; 56(6): 3092–3100. 10.1128/AAC.05904-11 22430975 PMC3370732

[47] Gibreel TM, Upton M. Synthetic epidermicin NI01 can protect *Galleria mellonella* larvae from infection with *Staphylococcus aureus*. J Antimicrob Chemother. 2013; 68: 2269–2273. 10.1093/jac/dkt195 23711896

[48] Matsuzaki T, Shimizu Y, Yokokura T. Augmentation of antimetastatic effect on Lewis lung carcinoma (3LL) in C57BL/6 mice by priming with *Lactobacillus casei*. Med Microbiol Immunol. 1990; 179: 161–168. 2144889 10.1007/BF00202393

[49] Matsuzaki T, Yamazaki R, Hashimoto S, Yokokura T. The effect of oral feeding of *Lactobacillus caei* strain Shirota on immunoglobulin E production in mice. J Dairy Sci. 1998; 81: 48–53. 9493081 10.3168/jds.S0022-0302(98)75549-3

[50] Segawa S, Nakakita Y, Takata Y, Wakita Y, Kaneko T, Kaneda H, et al Effect of oral administration of heat-killed *Lactobacillus brevis* SBC8803 on total and ovalbumin-specific immunoglobulin E production through the improvement of Th1/Th2 balance. Int J Food Microbiol. 2008; 121: 1–10. 18055049 10.1016/j.ijfoodmicro.2007.10.004

[51] Sashihara T, Sueki N, Ikegami S. An analysis of the effectiveness of heat-killed lactic acid bacteria in alleviating allergic diseases. J Dairy Sci. 2006; 89: 2846–2855. 16840600 10.3168/jds.S0022-0302(06)72557-7

[52] Lin W-H, Yu B, Lin C-K, Hwang W-Z, Tsen H-Y. Immune effect of heat-killed multistrain of *Lactobacillus acidophilus* against *Salmonella typhimurium* invasion to mice. J Appl Microbiol. 2007; 102: 22–31. 17184316 10.1111/j.1365-2672.2006.03073.x

[53] Ishikawa H, Kutsukake E, Fukui T, Sato I, Shirai T, Kurihara T, et al Oral administration of heat- killed *Lactobacillus plantarum* strain b240 protected mice against *Salmonella enterica* Serovar Typhimurium. Biosci Biotechnol Biochem. 2010; 74: 1338–1342. 20622449 10.1271/bbb.90871

[54] Villena J, Barbieri N, Salva S, Herrera M, Alvarez S. Enhanced immune response to pneumococcal infection in malnourished mice nasally treated with heat-killed *Lactobacillus casei*. Microbiol Immunol. 2009; 53: 636–646. 10.1111/j.1348-0421.2009.00171.x 19903264

[55] Vintiñi EO, Medina MS. Host immunity in the protective response to nasal immunization with a pneumococcal antigen associated to live and heat-killed *Lactobacillus casei*. BMC Immunol. 2011; 12 (46): 1471–2172. 10.1186/1471-2172-12-46 PMC3169484 21834957

[56] Maeda N, Nakamura R, Hirose Y, Murosaki S, Yamamoto Y, Kase T, et al Oral administration of heat-killed *Lactobacillus plantarum* L-137 enhances protection against influenza virus infection by stimulation of type I interferon production in mice. Int Immunopharmacol. 2009; 9: 1122–1125. 10.1016/j.intimp.2009.04.015 19410659

[57] Kobayashi N, Saito T, Uematsu T, Kishi K, Toba M, Kohda N, et al Oral administration of heat-killed *Lactobacillus pentosus* strain b240 augments protection against influenza virus infection in mice. Int Immunopharmacol. 2011; 11: 199–203. 10.1016/j.intimp.2010.11.019 21095259

[58] Forestier C, De Champs C, Vatoux C, Joly B. Probiotic activities of *Lactobacillus casei rhamnosus*: in vitro adherence to intestinal cells and antimicrobial properties. Res Microbiol. 2001; 152: 167–173. 11316370 10.1016/s0923-2508(01)01188-3

[59] Ramos AN, Gobbato N, Rachid M, González L, Yantorno O, Valdez JC. Effect of *Lactobacillus plantarum* and *Pseudomonas aeruginosa* culture supernatants on polymorphonuclear damage and inflammatory response. Int Immunopharmacol. 2010; 10: 247–251. 10.1016/j.intimp.2009.11.007 19932196

[60] Beristain-Bauza SC, Mani-López E, Palou E, López-Malo A. Antimicrobial activity and physical properties of protein films added with cell-free supernatant of *Lactobacillus rhamnosus*. Food Control. 2016; 62: 44–51. 10.1016/j.fm.2016.10.024 27889150

