# Supplementary figures and images for: Investigating the Effect of Different Treatments with Lactic Acid Bacteria on the Fate of Listeria monocytogenes and Staphylococcus aureus Infection in Galleria mellonella Larvae
Source: PLoS One. 2016 Sep 12;11(9):e0161263. doi: 10.1371/journal.pone.0161263 (PMC5019373; doi:10.1371/journal.pone.0161263)

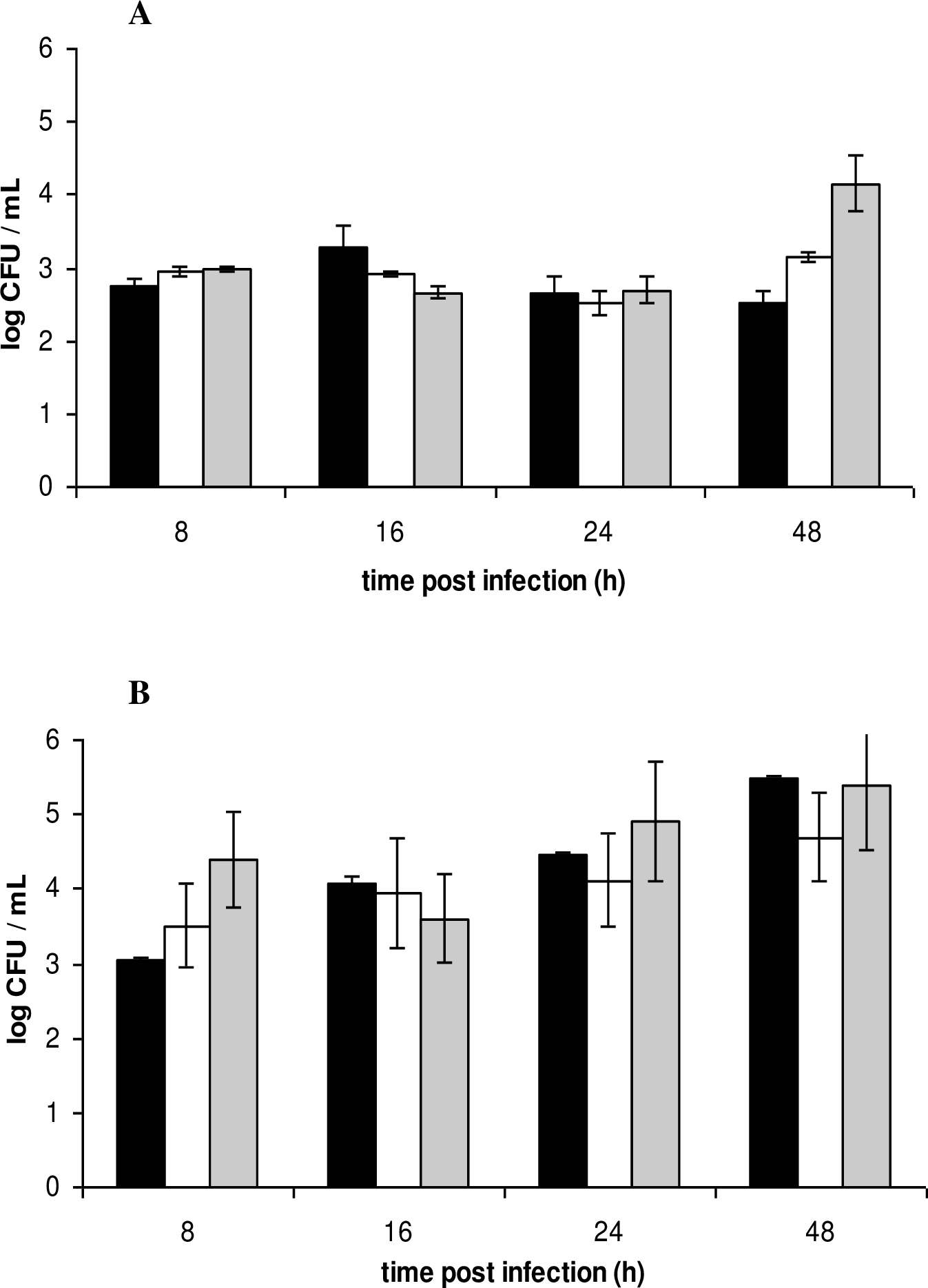

Supplement: S1 Fig — Larvae were injected with L. pentosus B281 (black bars), L. plantarum B282 (white bars) and L. rhamnosus GG (grey bars) at 6h (A) and 24h (B) prior to infection with the pathogen. (TIF) [file pone.0161263.s001.tif]

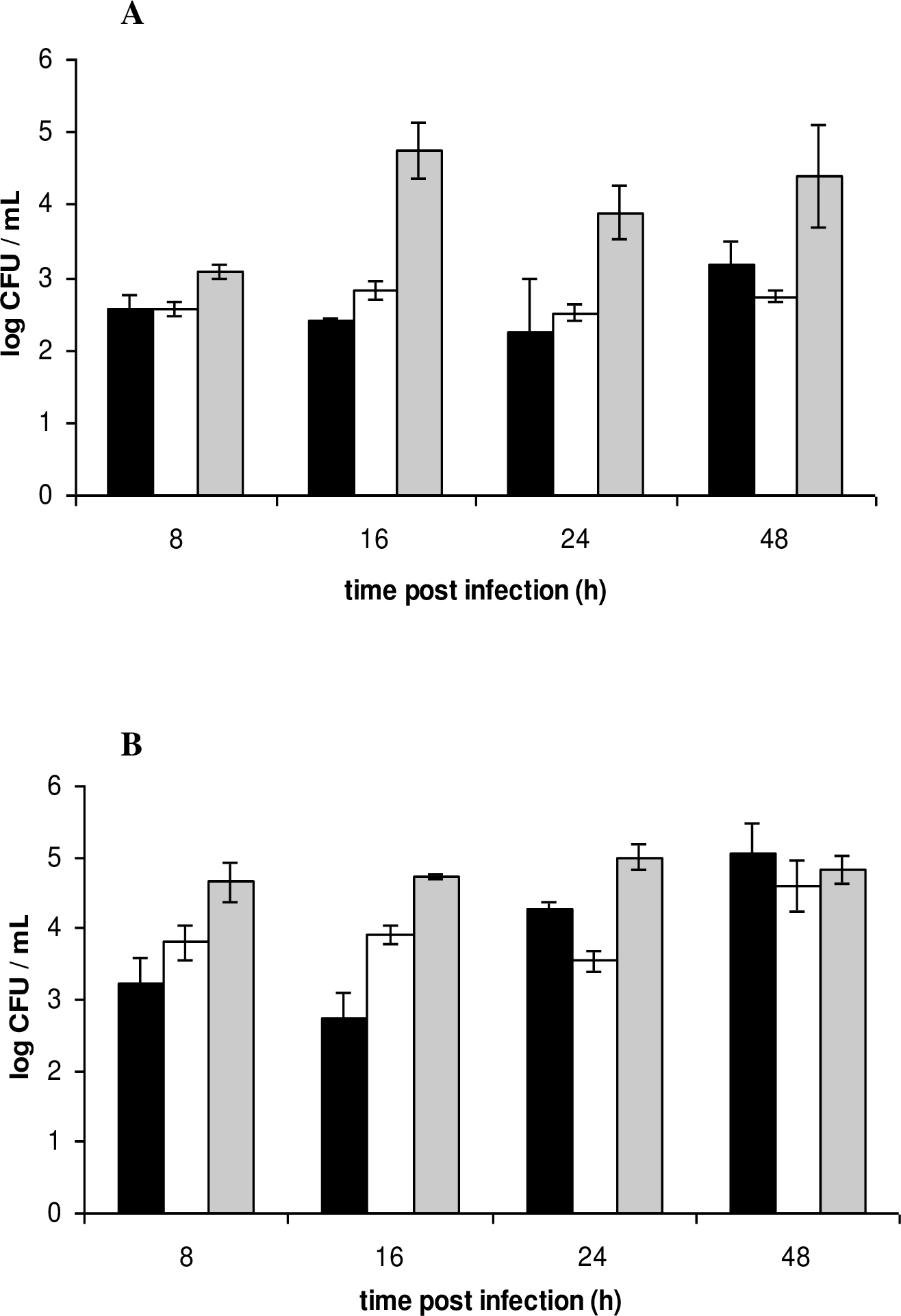

Supplement: S2 Fig — Larvae were injected with L. pentosus B281 (black bars), L. plantarum B282 (white bars) and L. rhamnosus GG (grey bars) at 6h (A) and 24h (B) prior to infection with the pathogen. (TIF) [file pone.0161263.s002.tif]
